# Supplementary material for: A Critical View of the Application of the APEX Software (Aqueous Photochemistry of Environmentally-Occurring Xenobiotics) to Predict Photoreaction Kinetics in Surface Freshwaters
Source: Molecules. 2019 Dec 18;25(1):9. doi: 10.3390/molecules25010009 (PMC7017383; doi:10.3390/molecules25010009)
Supplement: Supplementary file 1 [file molecules-25-00009-s001.zip › 3.2.4_gcc-4.4.0.zip!/3.2.4_gcc-4.4.0/3.2.4_gcc-4.4.0/doc/octave/html/interpreter/Structure-Arrays.html]

Structure Arrays - Untitled


Next: Creating Structures,
Previous: Basic Usage and Examples,
Up: Data Structures


---

#### 6.1.2 Structure Arrays

A structure array is a particular instance of a structure, where each of
the fields of the structure is represented by a cell array. Each of
these cell arrays has the same dimensions. Conceptually, a structure
array can also be seen as an array of structures with identical
fields. An example of the creation of a structure array is

```
     x(1).a = "string1";
     x(2).a = "string2";
     x(1).b = 1;
     x(2).b = 2;
```

which creates a 2-by-1 structure array with two fields. Another way
to create a structure array is with the `struct` function
(see Creating Structures). As previously, to print the value of
the structure array, you can type its name:

```
     x
          => x =
             {
               1x2 struct array containing the fields:
     
                 a
                 b
             }
```

Individual elements of the structure array can be returned by indexing
the variable like x`(1)`, which returns a structure with
two fields:

```
     x(1)
          => ans =
             {
               a = string1
               b =  1
             }
```

Furthermore, the structure array can return a comma separated list of
field values (see Comma Separated Lists), if indexed by one of its
own field names. For example

```
     x.a
          =>
             ans = string1
             ans = string2
```

Here is another example, using this comma separated list on the
left-hand side of an assignment:

```
     [x.a] = deal("new string1", "new string2");
      x(1).a
          => ans = new string1
      x(2).a
          => ans = new string2
```

Just as for numerical arrays, it is possible to use vectors as indices (see Index Expressions):

```
     x(3:4) = x(1:2);
     [x([1,3]).a] = deal("other string1", "other string2");
     x.a
          =>
             ans = other string1
             ans = new string2
             ans = other string2
             ans = new string2
```

The function `size` will return the size of the structure. For
the example above

```
     size(x)
          => ans =
     
               1   4
```

Elements can be deleted from a structure array in a similar manner to a
numerical array, by assigning the elements to an empty matrix. For
example

```
     in = struct ("call1", {x, Inf, "last"},
                  "call2", {x, Inf, "first"})
          => in =
             {
               1x3 struct array containing the fields:
     
                 call1
                 call2
             }
     
     in(1) = [];
     in.call1
          =>
            ans = Inf
            ans = last
```
